# Supplementary material for: Entangled Motifs in Membrane Protein Structures
Source: Int J Mol Sci. 2023 May 24;24(11):9193. doi: 10.3390/ijms24119193 (PMC10253074; doi:10.3390/ijms24119193)
Supplement: Supplementary file 1 [file ijms-24-09193-s001.zip › ijms-2402082-supplementary.pdf]

Entangled Motifs in Membrane Protein Structures

Leonardo Salicari [1,2] and Antonio Trovato [1,2,*]

1 Department of Physics and Astronomy 'Galileo Galilei', University of Padova, Via Marzolo 8, 35031 Padova, PD, Italy

2 National Institute of Nuclear Physics (INFN), Padova Section, Via Marzolo 8, 35131 Padova, PD, Italy

* antonio.trovato@unipd.it

Supplementary Figures

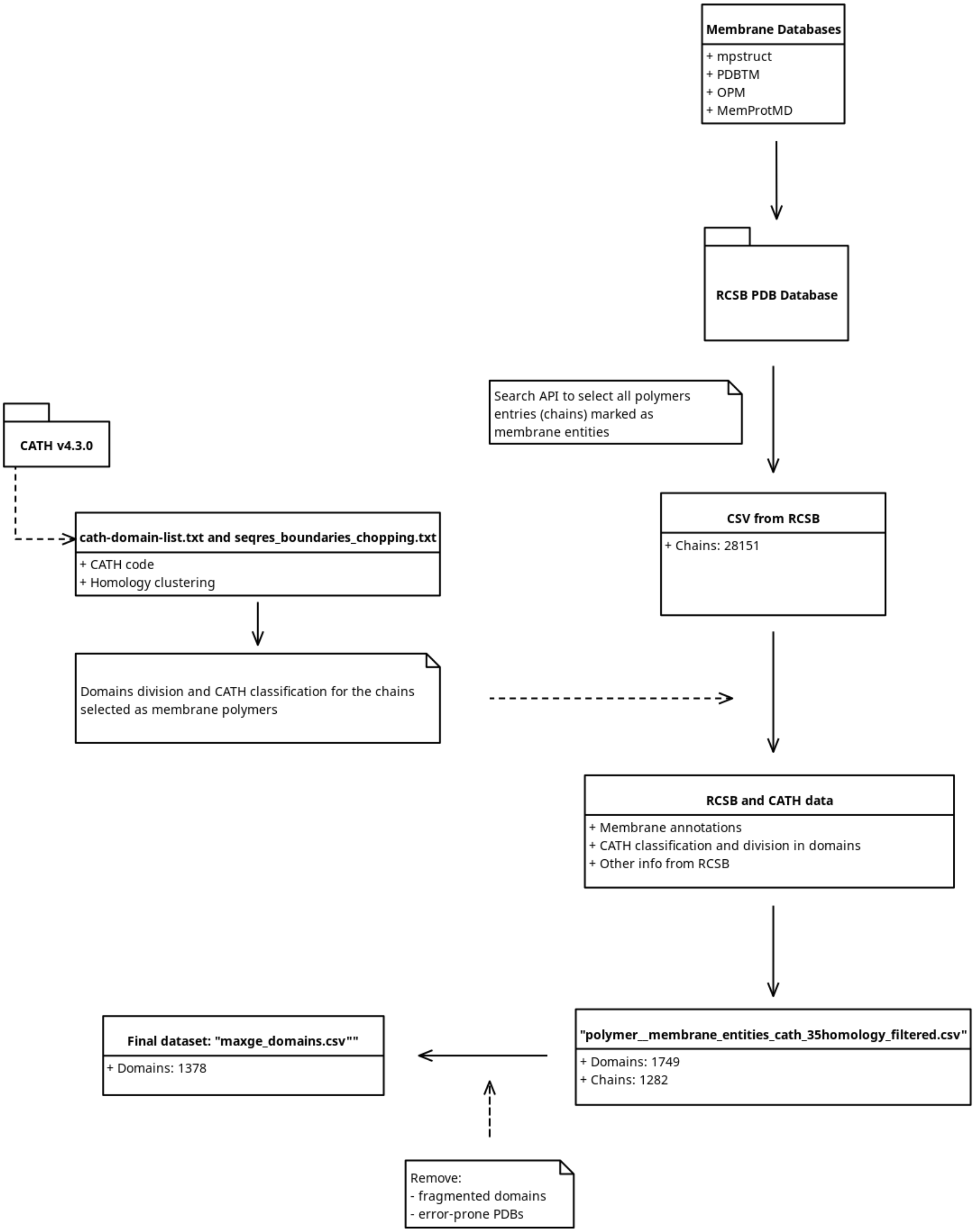

FIG. S1. **Pipeline for the generation of the data set of membrane protein domains.**
